# Challenges and opportunities for new intraoperative optical techniques in the surgical treatment of pituitary adenomas: a review

**DOI:** 10.1117/1.JBO.30.8.080901

**Published:** 2025-08-13

**Authors:** Félix Janelle, Victor Blanquez-Yeste, Trang Tran, Abdelhakim Khellaf, Romain Cayrol, Catherine Beauregard, André Lacroix, Alexander G. Weil, Philippe Lavigne, Frédéric Leblond, Moujahed Labidi

**Affiliations:** a University of Montreal, Division of Neurosurgery, Department of Surgery, Montreal, Quebec, Canada; b Polytechnique Montréal, Engineering Physics Department, Montréal, Quebec, Canada; c Centre de recherche du Centre hospitalier de l’Université de Montréal (CRCHUM), Montreal, Quebec, Canada; d University of Montreal, Montreal, Division of Pathology, Department of Medicine, Quebec, Canada; e University of Montreal, Division of Endocrinology, Department of Medicine, Montreal, Quebec, Canada; f University of Montreal, Division of Oto-rhino-laryngology, Department of Surgery, Montreal, Quebec, Canada

**Keywords:** pituitary adenoma, tissue optics, Raman spectroscopy, neurosurgery, clinical translation, instrumentation

## Abstract

**Significance:**

Surgery is a common intervention for patients with pituitary adenomas, particularly those experiencing endocrine symptoms or mass effect. Persistent challenges in pituitary surgery include the detection of small microadenomas, difficulty in discerning residual tumor from normal gland, and infiltrative adenomas. Although standard perioperative diagnostics include magnetic resonance imaging (MRI), computed tomography, ultrasound imaging, and neuronavigation, some centers employ intraoperative MRI, ultrasound, and fluorescence-guided endoscopy to increase the rate of gross total resection and preserve pituitary function. However, these techniques are often limited by availability, time requirements, cost, and inability to provide histological diagnosis.

**Aim:**

This review addresses opportunities to optimize both the extent of resection and gland preservation in pituitary adenoma procedures. We discuss the existing constraints faced in pituitary surgery and showcase the current and emerging detection techniques employed in clinical practice, as well as their limitations. We also discuss newer probing approaches such as elastography and Raman spectroscopy.

**Approach:**

We outline key attributes for an ideal optical tool, considering surgical theater functionality, ergonomics, and result reliability and accuracy.

**Results:**

A case study is presented describing the recent development of a fiber-optics instrument specifically designed for endonasal applications based on clinical requirements, along with preliminary data supporting the feasibility of intraoperative implementation.

**Conclusions:**

Current imaging and navigation tools, although invaluable, have inherent limitations in resolution, integration, and molecular specificity. Raman spectroscopy offers a promising, label-free method for real-time tissue identification, especially when integrated into fiber-optic probes for endonasal use. As a complementary tool, it could enhance intraoperative decision-making and surgical precision. Further clinical validation is needed to support its integration into standard workflows.

## 1 Introduction

The pituitary gland, located at the skull base within the sella turcica, controls several vital endocrine functions.^1^ It is composed of the anterior hypophysis (adenohypophysis) and the posterior hypophysis (neurohypophysis).^1^ Several key hormones are secreted by the anterior pituitary gland including growth hormone (GH), adrenocorticotropic hormone (ACTH), thyroid-stimulating hormone (TSH), gonadotropins (LH/FSH), and prolactin (PRL).^1^ These hormones, in turn, regulate the function of many organs including the adrenal glands, skin, ovaries and testicles, muscles and bones, uterus, mammary glands, thyroid, and kidneys.^1^ Each hormone is produced by a different cell line in the pituitary. These different cell lines can result in a variety of tumors that can originate from the pituitary gland, a heterogeneous group of lesions called pituitary adenomas/pituitary neuroendocrine tumors (PitNETs),^2^ which represent ∼15% of intracranial tumors.^3^^,^^4^ Many of these tumors remain asymptomatic, with cadaver studies showing that the population prevalence is higher than what is reported clinically.^5^ Symptomatic pituitary adenomas, either with systemic hormonal symptoms, through under- or overproduction, or local mass effect, are more common in women than in men.^2^

Pituitary adenomas can be clinically divided into two broad categories: functioning pituitary adenomas (∼2/3 of cases), which secrete hormones, and non-functioning pituitary adenomas (remaining ∼1/3 of cases).^2^ Most functioning adenomas secrete either PRL, GH, or ACTH, in decreasing order of frequency.^2^ The initial diagnosis is usually clinical or based on magnetic resonance imaging (MRI) findings and assessment of the patient’s hormonal function. A serum panel, including prolactin, TSH, free thyroxine (T4), ACTH, cortisol, GH, and insulin-like growth factor 1 (IGF-1), is obtained in all cases. In Cushing’s disease, salivary cortisol and 24-h urinary cortisol measurements are also performed, in addition to some “dynamic” tests such as a dexamethasone suppression test. In acromegaly, lack of suppression of GH following a 75 g oral glucose tolerance test (OGTT) is the gold standard diagnostic test, especially in cases when IGF-1 and GH are equivocal.^6^ The role of MRI is to search for a pathological lesion in the sellar region and to characterize the lesion’s morphology and relationships with adjacent structures. Pituitary adenomas are classified as microadenomas if they are <1  cm in diameter and macroadenomas if they are 1 cm or greater.^7^ Histopathological examination of the tumor specimen sampled during surgical resection remains the gold standard for diagnosis.^8^ As per the fifth World Health Organization (WHO) Classification of central nervous system tumors (2021), pituitary adenomas/PitNETs are clonal proliferations usually derived from one of the six recognized cell lineages, with a small proportion demonstrating incomplete differentiation or mixed hormonal expression, reflecting a wide range of histological subtypes.^8^ The pathologist classically analyzes the formalin-fixed paraffin-embedded (FFPE) tumor tissue microscopically, in conjunction with an immunochemistry panel including pituitary hormones and/or lineage-specific transcription factors, before making the diagnosis, which precludes immediate interpretation during the operation. Definitive histopathological analysis allows for a more precise categorization, aiding with clinical and biochemical correlation, e.g., in some adenomas presenting subclinical levels of secretion better characterized with immunohistochemistry. Frozen section analysis of tumor tissue during surgery can provide important diagnostic information in the context of suspected metastatic or inflammatory diseases. Studies have shown that intraoperative diagnosis using frozen section and/or cytological smear/touch imprint presents high concordance rates (up to 90%–95%) with the definitive diagnosis in pituitary adenomas/neuroendocrine tumors.^9^^–^^11^ Furthermore, in the context of microadenoma and Cushing disease, intraoperative diagnosis increases rates of pathologic confirmation (i.e., microadenoma identification by a pathologist) but could lead to a waste of limited tissue.^12^

Surgical resection is the definitive therapeutic option in several cases of pituitary adenomas, including in patients with endocrine symptoms or local mass effect.^13^^,^^14^ The endoscopic endonasal transsphenoidal approach is preferred in most cases. The goal is to achieve gross total resection of the tumor while limiting manipulation of the surrounding structures.^15^ Residual tumor volume significantly influences the likelihood of recurrence, potentially requiring further treatment and increasing the risk of early mortality.^16^^,^^17^ Conversely, inadvertent resection of normal pituitary tissue can lead to hormonal imbalances, which may require lifelong endocrine management.^18^

Preoperative MRI provides visualization of the sellar cavity to localize structural abnormalities such as tumors and is used during the resection through surgical navigation platforms.^19^ However, intraoperative tissue movement and gradual tumor resection cause discrepancies with the preoperative images.^15^ In addition, the substantial heterogeneity in tumor size, subtypes, and growth patterns further complicates complete resection. These challenges underscore the need for reliable intraoperative detection methods to guide resection. To address this, various intraoperative diagnostic and imaging techniques have been developed over the years, including intraoperative MRI, fluorescence imaging, and ultrasonography.^20^^–^^23^ These modalities provide real-time assessment of tumor presence and location during the procedure. Such methods aim to enhance surgical accuracy, improve resection rates, and lower the risk of recurrence. However, although some require a significant amount of time and resources, others have yet to demonstrate sufficient clinical benefit to justify widespread adoption.^24^^,^^25^ Consequently, optimal patient outcomes are still reliant on surgical expertise, with the best results obtained in centers of excellence led by specialized surgeons with a high patient volume.^20^^,^^26^

This literature review discusses the surgical challenges associated with both functioning and non-functioning pituitary adenoma resections, including the difficulty of distinguishing tumor tissue from normal structures, which directly impacts surgical and postoperative outcomes. The advantages and limitations of the various intraoperative imaging and detection techniques developed to address these challenges are assessed. Subsequently, the expected attributes required for a new optical tool to effectively overcome these limitations are discussed, focusing on the functional and ergonomic considerations within the surgical setting, as well as the reliability and accuracy of the detection results it should provide. Finally, a case study is presented, describing the recent development of a dedicated Raman spectroscopy probe adapted for endonasal surgical applications, enabling *in situ* tissue interrogation, while also comparing it to existing detection techniques.

## 2 Clinical Limitations in the Treatment of Non-functioning Pituitary Adenomas

**Fig. 1:**
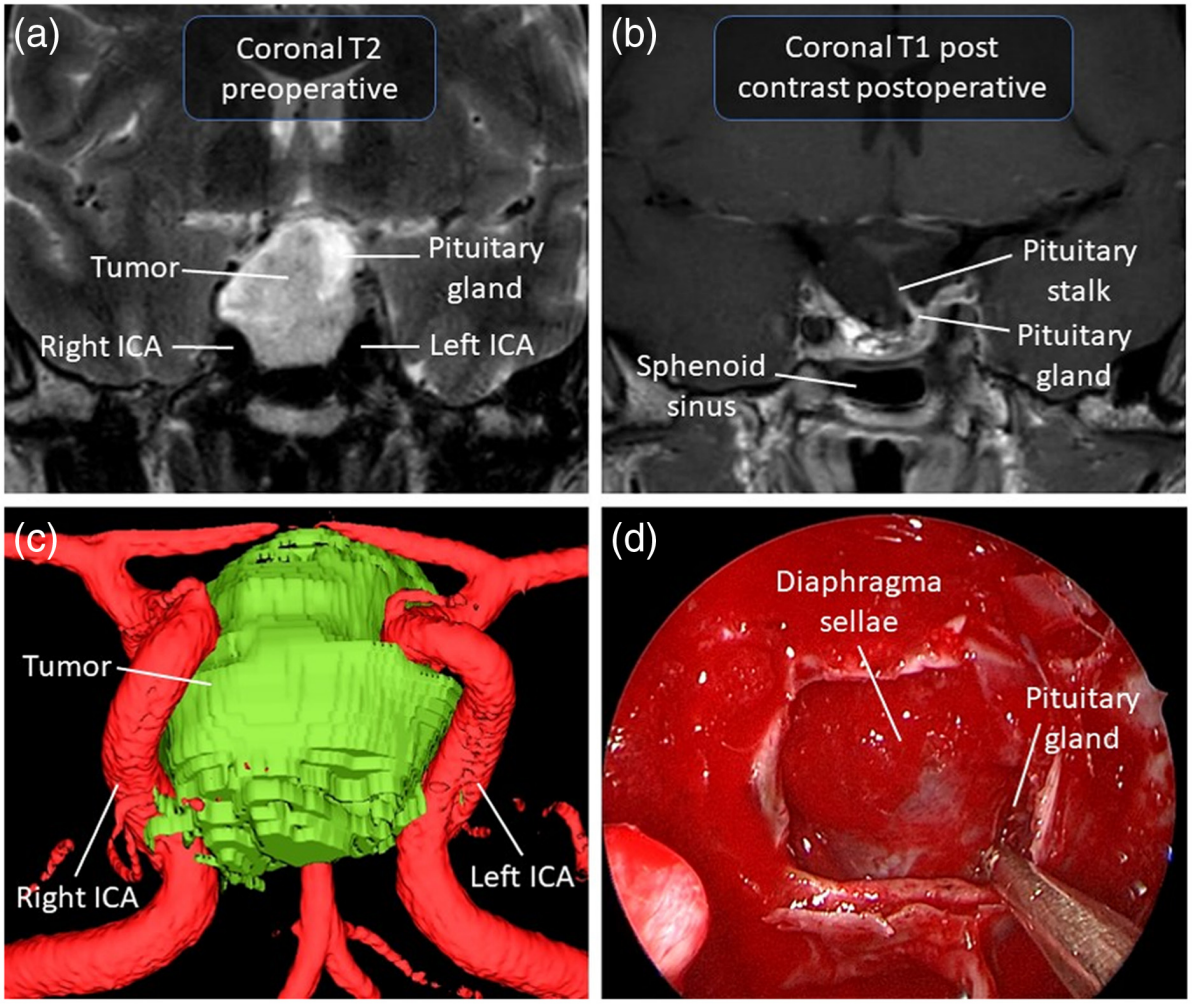
Illustrative case of a 40-year-old man with a pituitary macroadenoma found incidentally. There was radiological chiasmatic compression without signs or symptoms of optic neuropathy. (a) Preoperative T2-weighted MRI in coronal view of the macroadenoma pushing the pituitary gland to the left. (b) Postoperative T1-weighted MRI with contrast in coronal view. (c) Anterior view of a 3D model of the macroadenoma on the surgical navigation system from the T1-weighted MRI with contrast. (d) Endoscopic endonasal view at the end of the resection showing the pituitary gland displacement to the left and free fat graft used for closure. The interface between the tumor and the gland was visualized, and the gland was preserved to avoid postoperative endocrine deficits. ICA, internal carotid artery.

Non-functioning pituitary adenoma is the term used to refer to all pituitary adenomas that are not hormonally active clinically. In other words, they are pituitary adenomas that are not associated with clinical syndromes related to hormonal dysregulation, such as acromegaly, Cushing’s syndrome, hyperprolactinemia, or hyperthyroidism.^27^ Among all pituitary adenomas, 15%–30% are considered non-functioning.^28^^–^^30^ The absence of hormonal symptoms often prevents early diagnosis. The diagnosis is therefore often made either in the context of a local mass effect linked to the growth of a macroadenoma or as an incidental finding during brain imaging performed for another reason.^31^^,^^32^ When treatment is required, the first line of therapy is surgery, partly because there is no effective medical treatment,^33^ although dopamine agonists are sometimes used in this context with the hope of stabilizing lesion volume.^34^ The main indication for surgery in non-functioning pituitary adenomas is visual impairment/loss, as the optic chiasm is located superior to the pituitary gland. The level of urgency to operate depends on the severity of visual impairment. Duration as well as the severity of the visual field defect upon the patient’s presentation correlates inversely with the recovery of visual symptoms. Symptomatic improvement is reported in 80% to 90% of cases up to 1 year post-surgery.^33^ Other surgical indications include tumor growth on serial imaging, intractable headaches, and a large adenoma in an otherwise healthy young patient. Figure 1 presents the surgical case of a 40-year-old man from our institution in whom a macroadenoma was discovered incidentally.

Occasionally, tumor resection may be incomplete, resulting in insufficient visual apparatus decompression. In other circumstances, the resection is complete but is associated with damage to the normal pituitary gland, which can be manipulated excessively if not adequately identified and preserved intraoperatively. This may result in postoperative complications. The most common complication of transsphenoidal pituitary surgery is arginine vasopressin deficiency (AVP-D; previously known as central diabetes insipidus). In a series of more than a thousand operated cases, 16% of patients presented signs of early postoperative AVP-D.^35^ However, this postoperative condition is temporary in most cases; nonetheless, up to 12.5% of patients have permanent AVP-D after the procedure.^18^ In another series of 1571 cases of transsphenoidal surgery for pituitary adenoma, 34% of patients developed polyuria on the first postoperative day. Among these, 24% required vasopressin treatment.^36^ Persistent AVP-D was reported at 3 months in 0.9% and at 1 year in 0.25% of patients in this study.^36^ Postoperative anterior pituitary dysfunction is another potential complication following surgery, occurring in ∼9% of cases from a recent case series of 94 patients.^37^ The main surgical challenge that an intraoperative detection technology could help address resides in establishing a clear and unequivocal distinction of normal pituitary tissue from tumor. This distinction can be made for adenomas with a well-defined interface between the pathological and the healthy tissue. For tumors of a more infiltrative nature, the tumor margin will rather be a gradient of decreasing infiltration. Establishing this distinction would allow to leave intact the residual normal pituitary gland, thus preserving pituitary function, while achieving complete adenoma resection. In turn, this would reduce the risk of tumor recurrence and possibly eliminate the need for subsequent interventions.

One factor that may contribute to incomplete tumor resection is pituitary adenomas’ texture (i.e., stiffness), with most lesions being soft to the touch. However, it is estimated that 5% to 13% of these tumors are fibrous lesions.^38^ A fibrous tumoral consistency negatively affects the extent of tumor resection^39^ and is associated with a greater risk of developing postoperative hormone deficiencies,^40^ leading to higher surgical morbidity and mortality in affected patients.^38^ For all these reasons, the radiological prediction of tumor texture has been studied to better discern higher risk fibrous lesions.^39^^,^^41^^,^^42^ However, tumoral texture remains difficult to predict with preoperative imaging. Without a clear preoperative prediction of tumor consistency and heterogeneity, the surgeon may falsely interpret a change in texture as being the interface between the gland and the tumor, with a possible dramatic impact on the extent of resection. Intraoperative confirmation of the presence of the tumor would prevent this false interpretation of the tumor-gland interface and allow the surgeon to better navigate tumor heterogeneous zones *in vivo*.

When the surgical indication is local mass effect due to the adenoma, the surgeon also wants to establish if the suprasellar decompression is sufficient at the end of surgery. Typically, after tumor resection, the diaphragma sellae (sheet of dura mater) and/or the normal pituitary gland can be identified.^43^ Very often, there is also a degree of intrasellar herniation of the diaphragma sellae, correlating with the preoperative suprasellar extent of the tumor.^44^^,^^45^ This is a strong indicator of adequate optic apparatus decompression. Also, if the normal pituitary gland can be identified clearly, in most cases, this means the optic decompression is sufficient and the tumor resection is complete. This requires a visual assessment of the surgical cavity to identify the diaphragma sellae and gland, which comes with its share of uncertainty. A complementary intraoperative modality could address this limitation by providing confirmation of pituitary gland presence beyond visual inspection alone, enhancing the surgeon’s confidence in achieving adequate decompression.

## 3 Clinical Limitations in the Treatment of Functioning Pituitary Adenomas

The resection of functioning pituitary adenomas presents distinct surgical challenges from non-functioning adenomas, which could also benefit from intraoperative detection methods, such as optical techniques. In functioning pituitary adenomas, the main clinical issue is frequently hormonal; such tumors do not have to be large to require treatment.^46^ Secreting microadenomas can therefore cause systemic symptoms without causing symptoms due to local compression of surrounding structures from excessive growth. The clinical phenotypes are determined by the type(s) of hormones secreted in excess. The most common clinical subtype of functioning adenoma is a prolactinoma, typically arising from the PIT1 cell lineage, leading to central hyperprolactinemia (excess prolactin secretion), with characteristic features including galactorrhea and hypogonadism.^2^ If the tumor produces ACTH in excess, the patient will develop Cushing’s disease.^47^ In a patient with an adenoma hypersecreting growth hormone alone, acromegaly/gigantism progressively ensues.^48^ In all such cases, prompt treatment is required to lower associated mortality and morbidity.^46^

**Fig. 2:**
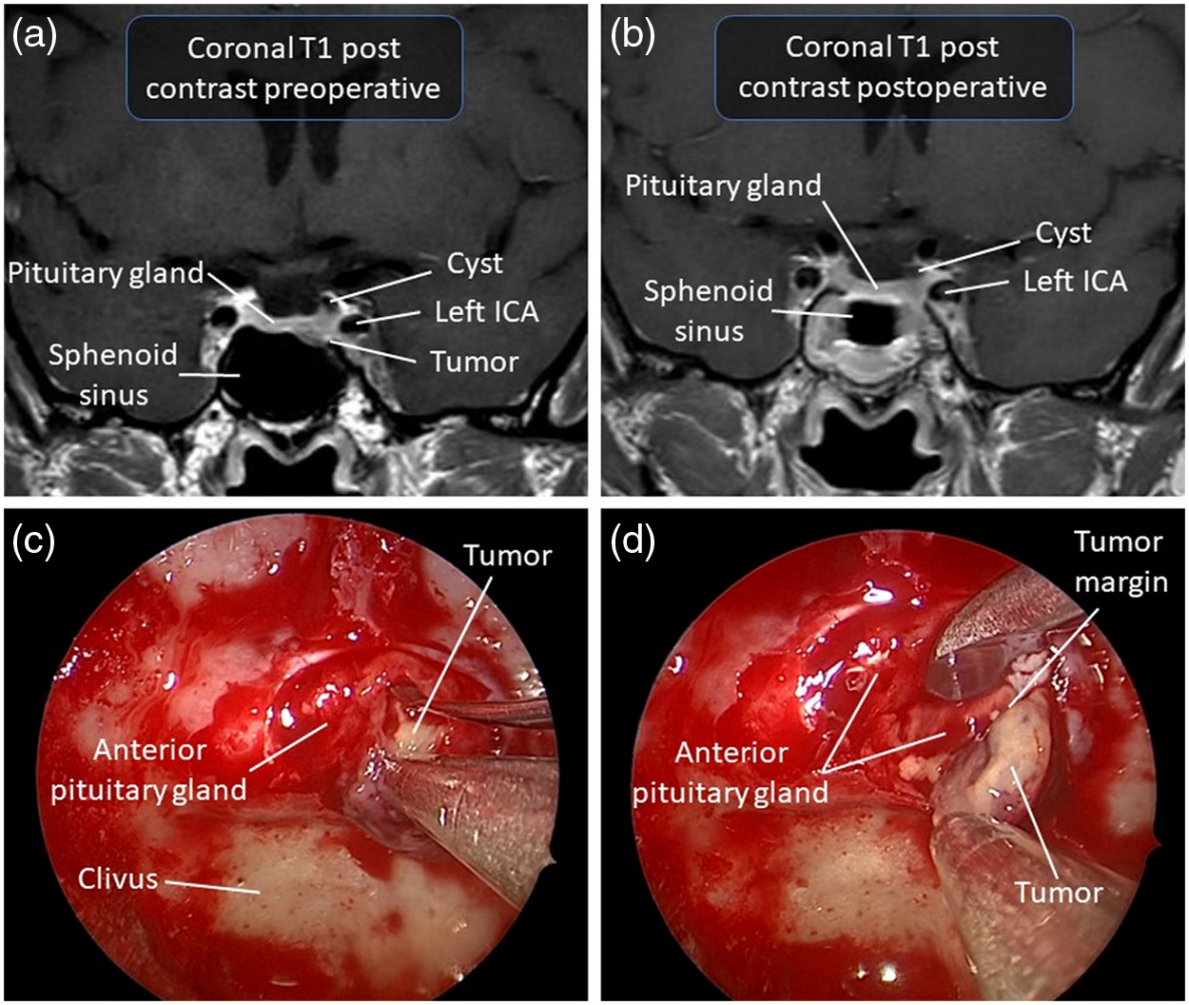
Illustrative case of a 57-year-old woman presenting with acromegaly. The patient was referred to neurosurgery for a pituitary microadenoma secreting growth hormone. (a) Preoperative T1-weighted MRI with contrast in coronal view of the microadenoma. (b) Postoperative T1-weighted MRI with contrast in coronal view. (c) Endoscopic endonasal view of microadenoma excision in the pseudocapsular plane. (d) Visualization of the tumor margin during dissection. Histopathological analysis of the specimen confirmed the diagnosis of a growth hormone (GH)-secreting adenoma, and the postoperative course was notable for complete remission of the acromegaly. ICA, internal carotid artery.

Potential therapeutic avenues include medical treatment, transsphenoidal surgery, and radiotherapy. The first line of treatment for prolactinomas is generally medical therapy, usually with a dopamine agonist (e.g., bromocriptine, cabergoline), whereas for other functioning pituitary adenomas, surgery is favored as the first line.^46^ Figure 2 illustrates a patient who underwent surgical resection for a microadenoma causing acromegaly. Combining multiple treatments is common in this type of tumor, especially when surgery is not curative or clinical control cannot be attained using medical treatment alone.

### 3.1 Detecting and Confirming Resection of Microadenoma in Normal Pituitary Gland

Surgery is a cornerstone in the treatment of patients with functioning microadenomas. It is very important to identify and remove the tumor completely, if feasible, in these cases. When the tumor is resected completely, evidence of disease remission is generally found immediately postoperatively. Such a “biochemical cure” is the primary aim of surgical resection and is associated with improved survival, especially in adenomas causing Cushing’s disease or acromegaly.^49^^,^^50^

Certain key challenges often preclude the complete removal of these tumors, including adequate preoperative radiological tumor detection and intraoperative confirmation of adenoma resection. As for all pituitary adenomas, MRI has been the imaging modality of choice since the 1990s for the identification of these smaller tumors.^26^^,^^51^ Despite decades of advances in medical imaging, some microadenomas are so small that their detection remains difficult. Conventional clinical MRI devices have a field strength of 3 Tesla, which allows for submillimeter resolution leading to voxel dimensions of 0.4 to 1 mm.^52^ Nearly 50% of microadenomas are diagnosed at 5 mm or less.^53^ A microadenoma of a few millimeters will therefore leave a radiological signature on only a few voxels, making its preoperative radiological assessment limited. Radiomic analysis has developed in recent years for pituitary adenomas. Through radiomics, advanced computational techniques allow for the extraction of more information by analyzing the spatial distribution of signal intensity and inter-voxel relationships.^54^ However, their scope remains limited for microadenomas as the issue of small size persists.

Particularly in cases of central Cushing’s disease, the diagnosis can be made even in the absence of a clearly defined pituitary adenoma on MRI. In such cases, inferior petrosal venous sampling is required to confirm the diagnosis. This challenge of not knowing precisely the presumed lesion’s location within the pituitary gland also arises when the anatomy is modified following a first surgery, and disease persistence or recurrence supports the need for reoperation.^55^^,^^56^ In all cases, the preoperative radiological information can be insufficient, and the surgeon must be guided by information available during the surgery, including the tumor’s visual appearance and texture.

Typically, the surgeon will make incisions in the gland to search for the underlying microadenoma, which should have a different consistency and color than the normal pituitary gland. This method, even when performed by experienced surgeons, is conducive to incomplete resections or involuntary resection of healthy tissue. In these cases, resection of part of the healthy gland is generally preferable to incomplete tumor resection because it allows for the treatment/cure of the adenoma. When the adenoma is suspected to be located on one side of the gland, an ipsilateral hemi-hypophysectomy can be considered, especially in persistent or recurring Cushing’s disease cases. Certain case series of microadenoma resections have reported postoperative hypopituitarism as the most frequent complication.^57^ No intraoperative tool is currently widely used and capable of distinguishing secreting pituitary adenomas from the healthy gland.

Uncertain or challenging radiological or intraoperative localization of a microadenoma results in more pituitary gland manipulation during surgery. If this is the case for a single adenoma, then the difficulties are increased for a double pituitary adenoma. Double pituitary adenoma is a condition characterized by the presence of two distinct adenomas in the pituitary gland, each coming from a separate clone with its own immunohistochemical signature.^58^^,^^59^ This condition is rare, with few cohorts of patients reported in the literature.^60^^–^^62^ Two tumors can sometimes be detected with MRI.^59^ In such cases, the neurosurgeon usually decides to carefully explore the pituitary gland by employing combined incisions of the gland, generally still using visual inspection for distinction between the pathological tissue of the adenoma and the healthy gland. This strategy is problematic considering that direct visualization can be impeded by bleeding, lesion size, and endoscope quality.

Another limitation of currently available clinical methodologies is the postoperative determination, with histopathological analysis, that the pituitary adenoma was in fact ACTH- or GH-immunoreactive, albeit “clinically silent.” In fact, it is recognized that cyclical hormonal production or low levels of hormonal production make the preoperative diagnosis of Cushing’s disease or slowly progressing acromegaly more challenging. Intraoperative determination of hormonal secretion by the adenoma could modify the surgical team’s approach allowing it to consider a more radical resection.

Recent surgical series have reported cases of microadenomas identified radiologically but difficult to visualize during operative resection and that persist on postoperative MRI.^63^ This highlights the complexity of resecting a microadenoma that can visually blend into the pituitary gland.^52^ Indeed, in transsphenoidal resection of adenoma for Cushing’s disease, incomplete tumor resection leads to persistence of the disease in ∼22% of patients.^54^^,^^64^ The prevalence of Cushing’s disease recurrence following pituitary surgery is ∼14%.^16^ Approximately 50% of related relapses occur during the first 50 months after initial surgery.^65^ It is for this specific reason that annual monitoring is recommended.^16^ The average recurrence rates for prolactinomas and GH-secreting adenomas are 11% and 3%, respectively.^66^ The first line of treatment for recurrent pituitary-related central Cushing’s disease is a second pituitary surgery with a mean remission rate of 64%.^16^ Success rates of second surgeries appear lower than first surgeries; medical therapy is therefore often considered in patients with tumor relapse or incomplete initial resection. ^67^

### 3.2 Tumoral Invasion of Surrounding Compartments

Growth of pituitary adenomas can be described either as expansion or infiltration of surrounding parasellar tissues. The integrity of the sellar dura mater and the intrasphenoidal septations can direct adenoma extension by promoting its growth towards paths of least resistance.^68^ Intrinsic tumor characteristics also guide its growth pattern. Slow and expanding growth from a tumor with a slow replication phenotype leads to a mass that exerts localized supraphysiological pressure. This change displaces healthy surrounding tissue, including the residual pituitary gland itself and surrounding structures. On imaging, an “expanding” pituitary adenoma classically shows well-defined tumor borders. Some anatomical regions are more prone to displacement while others are not, such as the bony part of the sella turcica. In some cases, it is possible to distinguish a so-called *pseudocapsular* *plane* in which the adenoma can be dissected.^69^ When such a plane can be found, it facilitates the preservation of normal adjacent structures and complete tumor resection, providing optimal results in terms of biochemical cure.^70^

As opposed to tumor expansion, infiltration is a different growth mechanism that generates distinct repercussions. These include penetration, incorporation, and destruction of adjacent tissues. An “infiltrating” tumor therefore usually shows poorly demarcated margins, with a corresponding radiological appearance. Up to 35% of pituitary adenomas are infiltrative, with risk increasing with tumor size (highest rate in macroadenomas).^3^^,^^71^ The remainder of this section will consider the surgical limitations encountered depending on the structures invaded by adenoma growth.

From a surgical point of view, infiltrating tumors of the pituitary gland invade the adjacent compartments and a gross total resection depends on the resection of all infiltrated tissue. The main surgical challenge related to tumor infiltration lies in the surgeon’s intraoperative ability to accurately identify surrounding tissue invasion and assess whether the infiltrated tissue can be safely resected.

The first compartment that can be infiltrated by the tumor is the pituitary gland itself. It is essential for the surgeon to have a representation of the tumor-gland interface modeled as precisely as possible to the real biological interface. The surgeon’s visuospatial conceptualization of the tumor-gland interface is modeled preoperatively by imagery and modulated intraoperatively mostly by macroscopic visual cues, which can lead to uncertain interface representation. This therefore raises the question of whether additional tools could be implemented to support the surgeon in having the best possible representation of the tumor-gland interface.

A second key compartment that can be infiltrated by pituitary adenomas is the cavernous sinus. Large tumors, especially those with cavernous sinus invasion, have often been considered an obstacle to gross total resection.^72^ Cavernous sinuses are complex venous dural sinuses containing important neurovascular structures including the internal carotid artery and cranial nerves III, IV, V1 (ophthalmic division of the trigeminal nerve), V2 (maxillary division of the trigeminal nerve), and VI.^73^ This makes tumor resection of this anatomical space particularly challenging.^74^ The cavernous sinus is defined by four walls: medial, lateral, posterior, and superior.^75^ The medial wall of the cavernous sinus, being the lateral border of the sella turcica, varies in structural thickness or defects.^2^ Thus, a pituitary adenoma can invade, invaginate,^76^^,^^77^ or extend into the cavernous sinus.^78^^,^^79^ In such a case, the resection of these walls, mainly the medial wall, allows access to the cavernous sinus. This technique has provided an effective way to improve tumor visualization, leading to better resection and a significant increase in endocrine function remission.^74^^,^^80^^–^^82^ However, this procedure entails an increased surgical risk for the neurovascular structures within the area of exploration.

**Fig. 3:**
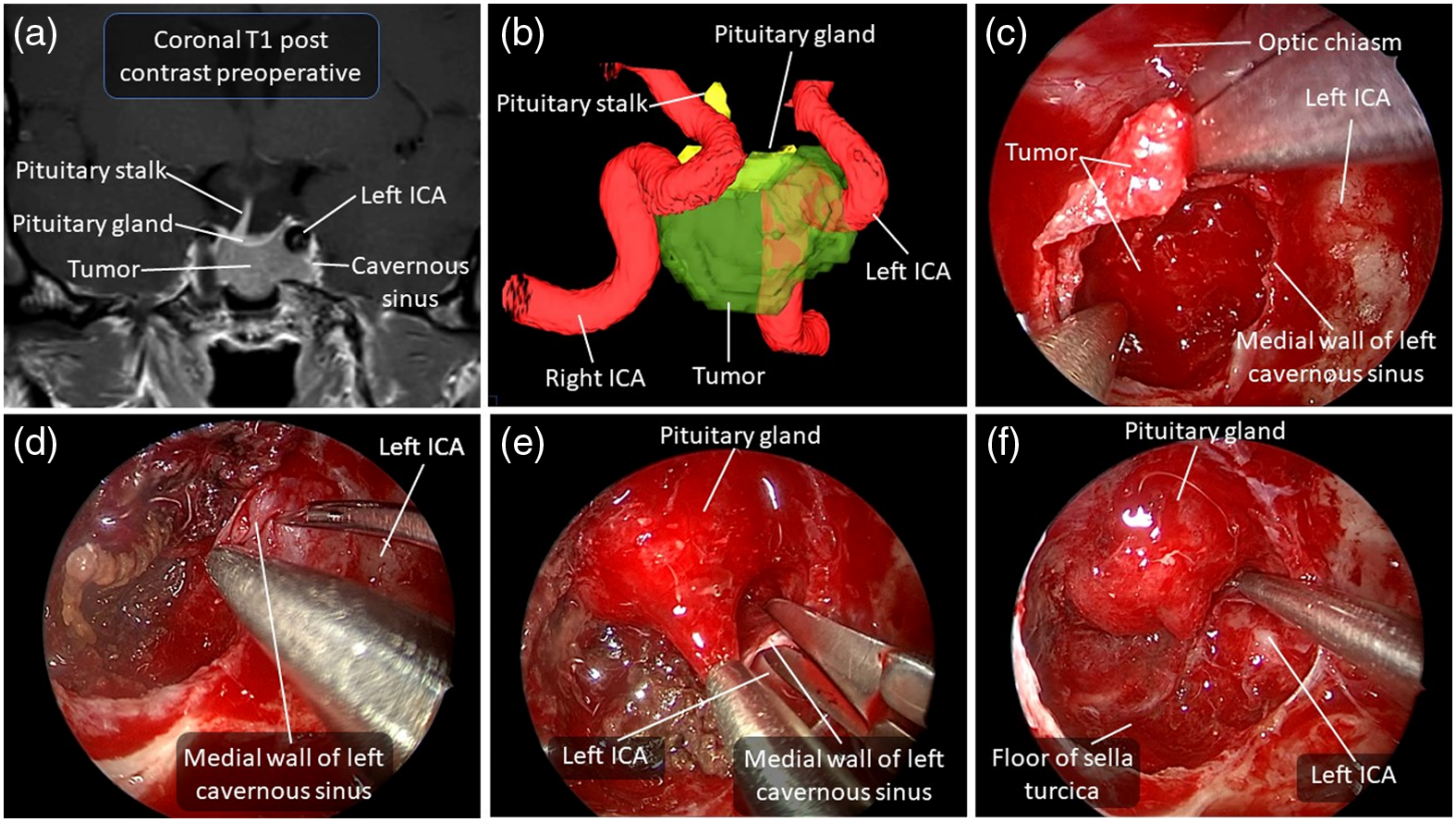
Illustrative case of a 36-year-old man referred for Cushing’s disease. (a) Preoperative T1-weighted MRI with contrast in coronal view showing a pituitary macroadenoma extending inferiorly and toward the left internal carotid artery. (b) Antero-lateral view of a 3D model of the macroadenoma in relation to the internal carotid arteries and pituitary gland. This model was made with the surgical navigation system from the T1-weighted MRI with contrast. (c) Endoscopic endonasal view of macroadenoma resection. (d)–(f) The bottom three images in the figure represent resection steps of the medial wall of the cavernous sinus due to radiological signs of cavernous sinus invasion. The medial wall incision of the cavernous sinus is performed (e), followed by its excision (e), then by post-excision visualization (f). Postoperative pathological analysis confirmed the tumor invasion of the cavernous sinus. Intraoperative confirmation of this tumor invasion would have provided an additional argument justifying excision of the medial wall of the cavernous sinus. The patient remains in remission as of the last follow-up (18 months postoperatively). ICA, internal carotid artery.

**Fig. 4:**
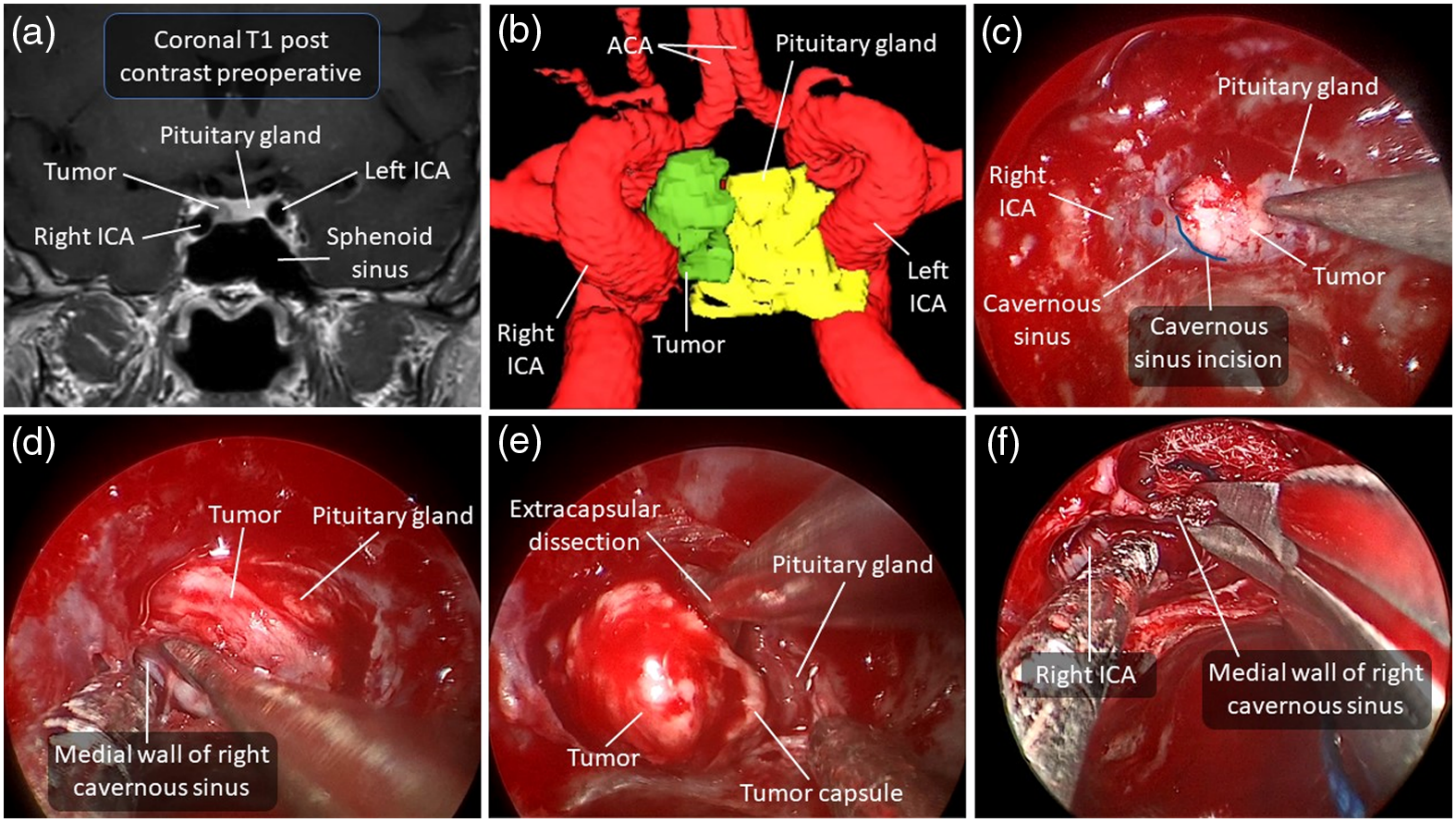
Illustrative case of a 69-year-old woman with a pituitary microadenoma causing acromegaly. (a) Preoperative T1-weighted MRI with contrast in coronal view of the microadenoma near the right cavernous sinus. (b) Anterior view of a 3D model of the microadenoma lining the medial wall of the cavernous segment of the internal carotid artery. This model was made with the surgical navigation system from the T1-weighted MRI with contrast. (c) Endoscopic endonasal view of the dura mater of the sella turcica. The tumor is visualized, and the planned cavernous sinus incision is marked in blue. (d) Visualization of the opening of the right cavernous sinus. (e) Extracapsular dissection of the tumor. (f) Excision of the medial wall of the right cavernous sinus. Pathology confirmed that the tumor was a pituitary adenoma that did not infiltrate the cavernous sinus despite its proximity. A tool allowing assessment of the presence of neoplastic cells intraoperatively would have made it possible to demonstrate the absence of infiltration of the cavernous sinus before its resection. ACA, anterior cerebral artery. ICA, internal carotid artery.

The Knosp classification is one of the most widely used radiological systems to determine the extent of cavernous sinus invasion by a pituitary adenoma, usually assessed on MRI.^83^ This classification is important for surgical planning and for predicting potential residual tumor post-resection.^84^ Low Knosp grades are predictive of better post-surgery remission rates, given that higher grades involve greater (gross/histological) invasion.^85^ Figures 3 and 4 show two examples of adenomas with imaging signs of cavernous sinus invasion. Pathological analyses confirmed that only the case shown in Fig. 3 had histopathological invasion of the cavernous sinus.

A third structure that can be invaded by an infiltrating adenoma is the sellar dura mater. This is of paramount surgical interest since invasion of the sellar dura mater has been shown to be a major determining factor in incomplete pituitary adenomectomy.^86^ Incomplete pituitary adenoma resection, occurring in ∼20% of primary pituitary tumor resections, increases the risk of recurrence. For these reasons, a surgeon will consider how far one should push the resection of the sellar dura and whether the dura of the floor of the sella turcica should be resected as well.

The fourth structure that can be invaded by an adenoma is the clival bone. A common belief was that adenomas rarely invaded the clivus. The basis for this thought arose from the paucity of reported cases, most of which involved ectopic pituitary adenomas.^87^^–^^89^ However, an analysis of clival invasion in a cohort of 390 patients with pituitary macroadenomas challenged the established paradigm by revealing that more than 8% of these tumors exhibited clival invasion.^90^ The surgical implications of the relatively high prevalence of bone infiltration are considerable. Indeed, for example, in some cases of invasive GH-producing macroadenomas causing acromegaly, there could possibly be a benefit in drilling the surrounding bone to reduce the tumor burden.

Determining which structures may be invaded is crucial due to the significant clinical implications of such invasions. It is important for a surgeon to identify tumor invasion of the different compartments to adjust their surgical approach accordingly. For all structures at risk, intraoperative confirmation of tumor invasion could further support surgical decision-making. A tool capable of distinguishing tumoral invasion from healthy structures could aid in preserving those critical tissues, including the normal gland, and in selecting cases in which a resection of the medial wall of the cavernous sinus should be considered. Intraoperative *in vivo* assessment of dura mater or bone invasion would make it possible to better justify and guide their resection, ensuring its necessity while minimizing its extent. Surgical approach correlates with clinical outcome, with disease control achieved in 60% to 90% of cases depending on the approach employed.^91^^–^^94^ The variability of these results is explained, among other things, by tumor volume, tumor subtype, previous surgery, and tumor extension into the cavernous sinus. In the event of invasion of the cavernous sinus, surgical exploration could improve cure rates. A 2022 meta-analysis reported that resection of the medial wall of the cavernous sinus could achieve disease remission rates up to 97% for a median follow-up of 30 months.^95^ Although 30 months is a relatively short time, these results are promising, considering the minimal morbidity reported from this additional surgical step.^95^ Similarly, optimizing the surgical approach can improve outcomes in cases of invasion of the clivus by a pituitary adenoma. In one clinical study, complete resection of the clivus was associated with a reduction in recurrences to 3% with a mean follow-up time of 29.5 months.^90^

## 4 Clinical Imaging and Detection of Pituitary Adenomas

### 4.1 Preoperative and Postoperative Imaging and Detection

As previously discussed, MRI is the modality of choice for identifying and characterizing functioning and non-functioning pituitary adenomas prior to resection. ^26^^,^^51^ A high-quality preoperative 3 Tesla MRI is paramount in preoperative planning and also used in postoperative evaluation. However, MRI at this conventional clinical field strength has limited sensitivity for detecting millimetric tumor volumes, such as microadenomas, due to its relatively coarse voxel size (0.4 to 2 mm).^52^ It may also fail to precisely identify the site of persistent or recurrent disease.^26^ Recent advancements in high-field MRI have enabled more detailed analysis of the tumor-pituitary interface by delivering stronger signals and enabling higher-resolution imaging with voxel sizes as small as 0.3 mm.^52^ In Cushing’s disease, 7 Tesla MRI has demonstrated superior detection of small tumors compared with weaker-field MRI.^96^ High-field MRI imaging enhanced the delineation of the tumor-pituitary interface, enabling the identification of previously undetected tumors.^97^ This modality was also superior in assessing tumor consistency^98^ and identifying neurovascular structures at risk near the surgical region of interest.^99^ Computed tomography (CT) has also been explored to visualize pituitary adenomas in preoperative planning. However, its effectiveness is limited by its inferior soft tissue contrast compared with MRI, which makes the latter the preferred modality.^26^

Meanwhile, molecular imaging modalities such as Positron Emission Tomography (PET), which uses radiotracers to image tumors by highlighting areas of increased metabolic activity, can be combined with MRI or CT to complement their anatomical imaging. Specific radiotracers, including C11-methionine, show promise by enabling localization of subgroups of adenomas, which are difficult to localize with MRI, such as *de novo*, persistent, or recurrent adenomas.^100^ However, significant heterogeneity in the uptake of the various radiotracers by different tumor subtypes has limited the use of this technology.^26^ PET also shares the coarse resolution limitation of MRI with respect to the pathology of interest,^101^^,^^102^ but the metabolic information it provides can help confirm tumor presence and assist in adenoma subtyping.^103^

Magnetic resonance spectroscopy (MRS) is another metabolic imaging modality that can complement MRI by providing biochemical information from large voxel volumes.^103^ Although it does not produce anatomical images, MRS has the potential to aid tumor confirmation and subtyping by analyzing metabolic profiles, with the added advantage of being more affordable than PET.^103^^,^^104^ It has also demonstrated potential for detecting markers of proliferation and hemorrhage in macroadenomas, although specific studies focusing on pituitary adenomas remain limited.^103^^,^^105^

In parallel, radiomics is emerging as another tool for enhancing pituitary adenoma diagnosis and characterization.^54^^,^^106^ By extracting and analyzing quantitative features from preoperative and postoperative imaging through machine learning techniques, radiomics has shown potential in differential diagnosis, subtype identification, consistency evaluation, invasiveness assessment, treatment response, and long-term outcomes prediction.^107^^–^^109^ However, its effectiveness is currently limited to macroadenomas, given the resolution constraints, and significant heterogeneity across tumor types, imaging protocols, and radiomics pipelines hinders reproducibility.^54^

Although imaging plays a crucial role in surgical planning and post-surgical evaluations, its influence on surgical decisions regarding tumor resection is limited by tissue movement and gradual tumor resection, which leads to discrepancies between the preoperative image and the real-time patient anatomy.

### 4.2 Intraoperative Detection and Imaging

To overcome this limitation and enhance real-time surgical guidance, intraoperative detection and imaging techniques have been developed and tested in operating rooms alongside current visualization and guidance systems, such as the endoscope and neuronavigation. The endoscope has significantly enhanced these procedures by virtually introducing the eye of the surgeon into the sphenoid and sellar cavity.^110^ In parallel, neuronavigation utilizes infrared cameras or electromagnetic sensors to track surgical instruments in real time and overlay their position onto 3D preoperative MRI or CT images.^19^^,^^111^ This system has been shown to improve resection rates, reduce postoperative complications, and allow operations that were previously judged as inoperable.^19^^,^^112^ However, it remains constrained by the limitations of the preoperative MRI it relies on, such as inconsistency with real-time anatomy.^113^ Neuronavigation provides spatial orientation but lacks real-time tissue differentiation, leaving surgeons dependent on their visual assessment and anatomical knowledge for decision-making.

#### 4.2.1 Anatomical intraoperative imaging

Intraoperative MRI (iMRI) was first described for transsphenoidal surgeries in 1994 to provide updated imaging and assess gradual tumor resection.^114^ Repeated scans during the operation have shown utility in detecting unexpected residual tumor and can increase the likelihood of achieving a gross total resection by up to 40%, with an even greater impact in secondary surgeries.^115^^–^^118^ However, iMRI is costly to install, as it requires specialized shielded surgical suites, which have limited its widespread use.^119^ In addition, it is estimated that the surgical workflow is typically interrupted for 30 min to obtain each scan, overextending overall operating time and anesthesia.^117^^,^^119^ Intraoperative CT (iCT) has also been explored for live surgical imaging, showing potential to increase the extent of resection in large and giant pituitary adenomas while reducing the risk of injury.^120^ Similar to iMRI, it is costly, time-consuming, and difficult to integrate into a surgical room. It requires a lead-shielded operating theater and staff evacuation to minimize radiation exposure. The total scanning time required for each use is ∼10−12  min.^120^ Although less costly and a bit quicker than iMRI, iCT is associated with reduced resolution and administration of radiation doses, which has limited its clinical experimentation.^20^

#### 4.2.2 Ultrasound probes

Hand-held ultrasound probes offer a less bulky and more time-efficient alternative for intraoperative imaging with insertable instruments developed to aid tumor localization and improve operation safety.^121^^,^^122^ Although some early prototypes were too large for safe insertion,^123^ recent designs, such as a single-use, bayoneted, side-firing instrument that provide images of internal body structures, have shown potential in increasing gross total resection rate while decreasing blood loss and operative times.^23^ However, the research is sparse and further development should focus on large multicenter trials to prove its clinical utility.^23^ In addition, the spatial resolution of ultrasound probes is usually lower than iMRI, as it remains a trade-off with penetration depth, making it less suitable for visualizing millimetric tumor volumes and precise tumor interface delimitation. This relatively low resolution and the variability in image interpretation have contributed to a decline in the popularity of the technique over the recent years.^20^

#### 4.2.3 Fluorescence-guided endoscopy

Fluorescence-guided endoscopy enhances tumor contrast beyond that of white-light endoscopic imaging, offering potential to improve resection rates and overall survival.^124^ Several fluorophores have been investigated, each presenting unique advantages and limitations. 5-ALA, which leads to protoporphyrin IX accumulation, can help detect microadenomas that could not be seen on MRI, but its uptake varies across adenoma subtypes, particularly between corticotrophs, other functioning, and non-functioning adenomas.^15^^,^^125^ OTL38 and sodium fluorescein showed results that indicate potential for improved tumor visualization with high specificity but also showed limited sensitivity across adenoma subtypes due to tumor heterogeneity.^124^ Near-infrared fluorescence of indocyanine green (ICG), a technique widely used in vascular imaging, has demonstrated improved tumor contrast when combined with delayed imaging techniques.^22^ Furthermore, the second window ICG appears to be a promising option for use in all pituitary adenomas, regardless of their secretory status. However, its specificity is limited by its dependence on passive diffusion and accumulation through the enhanced permeability and retention effect.^22^^,^^124^

Challenges to the widespread use of fluorescence imaging for pituitary adenoma surgery include the limited clinical availability of most agents, as well as the technological and economic challenges to incorporating them into routine surgical flow.^124^ In addition, their time-sensitivity may not align with the demands of complex surgeries, and they require direct exposure of the structures, which is not always possible.^20^ Currently, only ICG-enabled endoscopes are commercially available for transsphenoidal surgery, and limited research prevents definitive conclusions about its potential to address clinical needs.^22^^,^^126^

#### 4.2.4 Emerging technologies

A recent study examined high-resolution contact-endoscopy to improve visualization, enabling the observation of microstructures through direct tissue contact. Although further validation in larger studies is necessary, preliminary results suggest it could help differentiate normal pituitary glands from adenomas.^127^ However, variations in vascularity among adenoma subtypes may impact its effectiveness. Another emerging technology stems from growing interest in studying the mechanical properties of human tissues at subcellular resolution, which has been fueled by evidence that cell-scale tissue mechanics influence tumor progression.^128^ Specifically, elastography has also been explored *ex vivo* to distinguish pituitary adenomas based on their micromechanical signature.^129^ Adenoma tissues were found to be softer and exhibited a more uniform pattern compared to healthy tissues across all adenoma subtypes, suggesting that this technology could be integrated into a handheld probe for *in vivo* interrogation.^129^

## 5 Discussion and Clinical Need for Intraoperative Surgical Guidance

Although anatomical imaging modalities such as MRI are indispensable for preoperative surgical planning, their intraoperative utility is currently constrained by acquisition time, cost, and the complexity of surgical integration.^117^^,^^119^ In addition, their spatial resolution restricts their ability to address key surgical challenges, including estimating residual tumor volume or localizing microadenomas.^26^^,^^119^ High-field MRI and molecular imaging techniques, such as PET, offer promising improvements for preoperative assessment and could serve as advanced intraoperative guidance tools in specialized surgical centers. Ultrasound probes and fluorescence-guided endoscopy provide more immediate imaging solutions. However, ultrasound is hindered by limited spatial resolution and inconsistent interpretation, whereas fluorescence-guided endoscopy faces challenges in clinical translation from *in vitro* studies to transsphenoidal procedures, including variable fluorophore uptake across tumor subtypes.^23^^,^^124^

Over recent decades, advancements in imaging techniques, surgical equipment, and operative strategies have led to higher resection rates and fewer complications.^20^^,^^130^ However, tumor heterogeneity in size, localization, shape, and nature has led to certain techniques being more effective for specific tumor types or cases and to the use of complementary techniques by multidisciplinary teams.^20^^,^^130^ Further refinement of this set of tools could enhance patient outcomes, yet a critical need remains for an effective intraoperative tumor detection instrument—particularly for millimetric tumor volumes, such as residual tumor, microadenomas, and infiltrative disease.

Localized tissue interrogation technologies could fill this gap by providing real-time molecular or structural information to complement imaging and overcoming its resolution limitation. Rather than replacing imaging, such tools could complement it by providing additional real-time information about the nature of targeted tissue regions. A key limitation of point-based methods is their inherently limited field of view, which may result in numerous measurements across the surgical field in cases of large tumors or diffusive borders. For clinical adoption, this modality must provide rapid measurements to avoid prolonging surgery while allowing repeated readings.

An ideal intraoperative diagnostic instrument would be minimally invasive and capable of accurately differentiating pituitary adenomas from normal gland tissue in real time, assisting in defining the tumor-gland interface for both functioning and non-functioning adenomas. Beyond tumor identification, distinguishing between adenoma subtypes, such as GH-, ACTH-, PRL-, and LH/FSH-secreting, would also be desirable. Furthermore, distinguishing the tumor from adjacent healthy structures, such as the arachnoid membrane, dura, and bone, could improve resection rates and help preserve normal function in cases of tumor invasion. Ideally, the tool would also provide an indication of the degree of infiltration, enabling the surgeon to assess the relevance of resecting a given structure. For example, quantifying the percentage of tumor infiltration relative to normal tissue within the sellar dura could directly influence the surgical approach. Finally, an additional desirable feature would be differentiating normal anatomical structures to provide general surgical guidance and enhance operation safety.

To achieve clinical integration, an insertable hand-held device must be designed for practical use within the constraints of endoscopic endonasal surgery. The instrument should have a long and bayoneted design, a standard feature for endonasal instruments. It facilitates maneuverability in restricted and deep operating fields and the collaboration of two surgeons, which is frequently the case in endonasal skull base surgery. A minimal distal working length of 10 to 12 cm is necessary to reach the posterior part of the sella turcica. In addition, the distal tip must be angled and/or malleable to increase the reach of the instrument down the long surgical corridor through simple rotation while also enhancing visualization of the point of contact. The proposed point-like interrogation tool should have a shallow depth of field (<1  mm) to resolve thin structures such as the dura or the medial wall of the cavernous sinus. Finally, compatibility with endoscopic systems is essential for seamless integration into the current surgical workflow.

Diagnostic performance is equally critical. The tool must provide accurate, reliable, and reproducible measurements under intraoperative conditions. To potentially influence surgical decisions, it should produce consistent diagnostic output when interrogating the same tissue region and maintain reliability despite operative field disturbances. Transsphenoidal surgery sometimes involves continuous blood oozing; therefore, the measurement should remain accurate and unaffected by the presence of blood. Clinically relevant benchmarks would include sensitivity and specificity above 90% for distinguishing adenomas from normal gland tissue (ideally compared with the surgeon’s diagnostic accuracy), inter-measurement variability below 5%, and robustness in the presence of the blood. Meeting these criteria is essential to integrate such a tool and fill a critical gap in the neurosurgical toolkit—enabling real-time, tissue-specific guidance during pituitary tumor surgery to improve resection rates, preserve function, and ultimately enhance patient outcomes.

## 6 Case Study: Raman Spectroscopy Probe for Endonasal Surgery

Raman spectroscopy is a label-free optical technique that analyzes inelastically scattered light to provide molecular information of tissues in the form of a spectrum. It detects the relative concentration of biochemical components such as proteins, specific amino acids, lipids, and nucleic acids using—as a surrogate—Raman peaks associated with specific vibrational bonds. This specificity enables molecular-based differentiation for a range of biomedical applications, including cancer detection. Raman spectroscopy-based studies investigated tumor detection of various organs, including brain,^131^^–^^133^ ovary,^134^ prostate,^135^^,^^136^ pancreas,^137^ breast,^138^^,^^139^ skin,^140^^,^^141^ lung,^142^^,^^143^ and metastatic sites.^144^^,^^145^

Its relevance to pituitary surgery has also been demonstrated with tumor detection on biopsies. In 2012, Raman analysis of several brain tumor specimens obtained during surgery, including pituitary adenomas, demonstrated potential for differentiation from normal brain.^146^ In 2019, Austrian researchers employed Line Scan Raman Microspectroscopy to identify pituitary adenoma subtypes with 95% accuracy for distinguishing adenomas from healthy glands and over 84% for subtype classification.^147^ This system was later incorporated into a multimodal optical imaging technique combining Raman spectroscopy with other modalities, which resulted in improved subtyping classification accuracies up to 99%.^148^

**Fig. 5:**
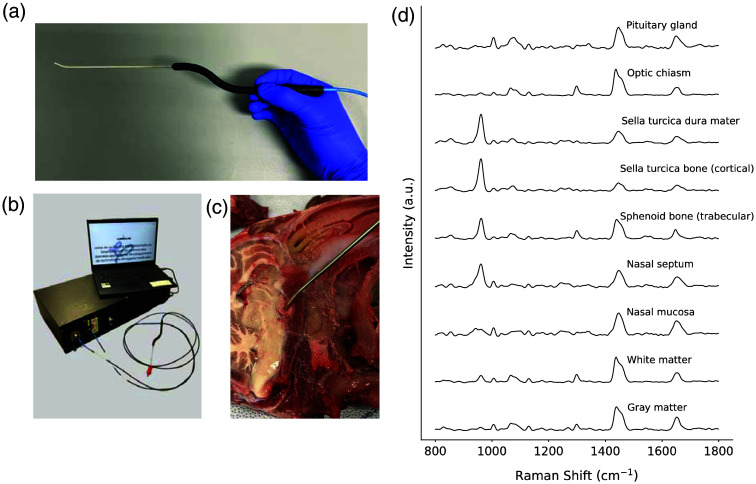
(a) Handheld Raman spectroscopy probe specifically designed for use during endonasal surgery. (b) Raman spectroscopy system equipped with the endonasal probe. (c) *Ex vivo* Raman spectroscopy measurement performed in situ on the pituitary gland of a lamb with the designed probe. (d) Average Raman spectra for each lamb tissue type, with corresponding variance computed for each spectral bin across measurements in light grey. Each spectrum is averaged on more than 20 different measurement locations per tissue type.

Although Raman analysis of biopsies could be valuable in a clinical setting, providing a faster alternative to histopathological analysis and retrospective validation, it does not support real-time surgical decision-making prior to tissue resection. To bridge this gap, intraoperative Raman probes have been developed to allow real-time tissue characterization in surgery. These instruments have been evaluated in multiple surgical fields including brain cancer,^149^ skin cancer,^150^^,^^151^ and lung cancer.^143^^,^^152^ Our group also developed an intraoperative single-point fiber optics probe system that can measure Raman spectra *in situ* within less than a few seconds [Fig. 5(b)]. The *in vivo* and in-human cancer detection capabilities of this probing system have been demonstrated in clinical studies involving gliomas, meningiomas, and metastases in the brain,^153^ as well as prostate tumors.^154^ Multiple other *ex vivo* human specimen studies have demonstrated cancer detection in the breast,^155^ lung,^156^ prostate,^157^ and ovary tissues.^158^ The system has also enabled the classification of different normal tissue structures, such as brain and gray matter,^159^ and major structures associated with the spinal cord, including the bone, muscle, adipose, cartilage, and spinal cord.^160^ The molecular specificity of the Raman spectroscopy makes it versatile and a strong candidate for the various differentiation needs of pituitary adenoma surgery outlined in Sec. 5, such as distinguishing between adenoma and normal gland, identifying tumor infiltration, and classifying surrounding anatomical structures.

The hand-held Raman probe of the system was adapted for transsphenoidal surgery with a bayonetted form factor for practical handling and a 30-deg angled tip for improved probing and visualization in the cavity [Fig. 5(a)], conforming to technical specifications set by surgeons (Sec. 5). In addition, the technology was made compatible with an endoscopic system. The infrared contribution of the endoscopic light illuminating the probe during measurements can induce white light artifacts in Raman spectra.^161^ The light source from the endoscope was thus retrofitted to include low-pass optical filters with a cutoff at 750 nm to ensure near-infrared components of the broadband white light source were eliminated. This was done to ensure Raman detection could be achieved without the requirement to shut the light source, ensuring the probe tip could be guided to its desired location under full bright light imaging.

A preliminary *ex vivo* study conducted on lamb heads demonstrated the feasibility of using the endonasal Raman system to acquire and differentiate *in situ* measurements from different intracranial structures commonly encountered during transsphenoidal surgery.^162^ These included the pituitary gland, the dura mater and bone of the sella turcica, nasal septum, and mucosa, as well as white and gray matter [Fig. 5(d)]. When combined with support vector machine (SVM) classification models, the system was able to differentiate spectra from these structures with an accuracy exceeding 95%. To demonstrate medical applicability, future work will involve clinical studies aimed at developing machine learning models for the detection of human pituitary adenomas and tumor invasion, as well as models capable of distinguishing the normal pituitary gland from adjacent anatomical structures. These models may also be trained on Raman spectra to estimate the percentage of tumor cells within the interrogated area to characterize tumor invasion and diffuse borders.

Although such probes are inherently limited by the small diagnostic surface interrogated per measurement, their high molecular specificity, rapid acquisition time, and demonstrated tissue classification capabilities position endonasal Raman spectroscopy systems as strong candidates for addressing the real-time diagnostic needs of pituitary surgery—including adenoma detection, subtype classification, assessment of tumor infiltration, and discrimination of adjacent anatomical structures.

## 7 Conclusion

In this paper, we explored the current surgical challenges in pituitary surgery, with a focus on the limitations encountered during the treatment of non-functioning and functioning pituitary adenomas. We reviewed current and emerging diagnostic techniques developed to support these procedures, including preoperative and postoperative imaging, their current development and intraoperative application, neuronavigation, ultrasound probes, fluorescence-guided endoscopy, and newer probing approaches such as elastography and Raman spectroscopy. Each modality contributes uniquely to the surgical workflow: imaging remains indispensable for planning and post-treatment evaluation, intraoperative navigation aids anatomical localization, and ultrasound and fluorescence imaging can provide real-time structural feedback. However, each is also limited in scope, whether due to complex clinical integration, spatial resolution, reliance on exogenous agents, or limited molecular specificity. This underscores the need for complementary approaches that enhance intraoperative decision-making without adding procedural complexity.

Among these emerging technologies, Raman spectroscopy stands out for its ability to provide label-free, real-time molecular characterization of tissue. Its integration into a fiber-optic probe system enables rapid and targeted *in situ* analysis without disrupting surgical workflow, making it well-suited for endonasal applications. Raman probes could help address a key unresolved limitation across current intraoperative methods: the reliable identification of small tumor volumes, such as functioning microadenomas or subtle residual tissue, that may evade detection by imaging or visual inspection.

The broader clinical value of Raman spectroscopy is gaining recognition across various surgical fields such as brain and skin surgeries. Although its capacity to distinguish intracranial parasellar structures *ex vivo* in an animal model has been demonstrated,^162^ its intraoperative use during pituitary surgery has yet to be explored. Recent technological developments, including our group’s adaptation of a Raman probe for endonasal use, aim to bridge this gap by enabling real-time molecular interrogation during pituitary surgery. Rather than replacing existing modalities, we suggest integrating Raman spectroscopy as a complementary probing tool. When integrated alongside imaging and navigational systems, Raman-based tissue identification could help address current limitations with localized interrogation and enhance precision in tumor margin delineation, subtype identification, and anatomical discrimination. Future studies should focus on validating this technology *in vivo* in clinical settings, with particular attention to developing robust and accurate classification models that address clinical needs and ensure seamless integration into the surgical workflow.

Overall, we believe that the development of targeted and localized probing modalities could complement the refinements of the suite of techniques currently available in transsphenoidal surgeries and further enhance patient outcomes. Probing could enable real-time diagnosis of small volumes during surgery, whereas advances in imaging resolution and molecular imaging could further improve preoperative planning, post-treatment evaluation, and overall treatment precision.

## Data Availability

Data and code are available from the authors upon reasonable request.
